# Swine CAFOs & Novel H1N1 Flu: Separating Facts from Fears

**DOI:** 10.1289/ehp.117-a394

**Published:** 2009-09

**Authors:** Charles W. Schmidt

**Affiliations:** **Charles W. Schmidt**, MS, of Portland, Maine, has written for *Discover Magazine*, *Science*, and *Nature Medicine*. In 2002 he won the National Association of Science Writers’ Science-in-Society Journalism Award

With cases documented in more than 170 countries, the global swine flu pandemic that erupted in spring 2009 remains a serious public health problem. Caused by a strain of H1N1 influenza virus, which is normally found in pigs, the flu now known as novel H1N1 has so far been less severe than regular seasonal flu in terms of deaths and hospitalizations. Yet given its remarkable capacity for human-to-human transmission and a widespread lack of immunity among potentially exposed people, it’s likely the number of cases will rise during the flu season later this fall and winter, according to many public health experts. Given that possibility, enormous resources are being mobilized to address novel H1N1, with an emphasis on vaccine development, education, and efforts to its limit its movements among human communities.

Yet one potential source of the original outbreak—factory swine farming in concentrated animal feeding operations (CAFOs)—has received comparatively little attention by public health officials. CAFOs house animals by the thousands in crowded indoor facilities. But the same economy-of-scale efficiencies that allow CAFOs to produce affordable meat for so many consumers also facilitate the mutation of viral pathogens into novel strains that can be passed on to farm workers and veterinarians, according to Gregory Gray, director of the Center for Emerging Infectious Diseases at the University of Iowa College of Public Health.

“When respiratory viruses get into these confinement facilities, they have continual opportunity to replicate, mutate, reassort, and recombine into novel strains,” Gray explains. “The best surrogates we can find in the human population are prisons, military bases, ships, or schools. But respiratory viruses can run quickly through these [human] populations and then burn out, whereas in CAFOs—which often have continual introductions of [unexposed] animals—there’s a much greater potential for the viruses to spread and become endemic.”

Gray says workers exposed routinely to livestock can pass these zoonotic infections—which transmit readily among humans and animals—on to the wider public. However, public health agencies that monitor risks from zoonotic infections routinely overlook CAFO workers, according to Ellen Silbergeld, a professor at the Johns Hopkins Bloomberg School of Public Health. And animal disease sampling data collected by the food animal industry typically are not shared publicly, according to Gray, although such data could reveal how novel pathogens evolve in CAFOs and how they might move among animals, workers, and the broader community. Experts believe that without these data, society has a diminished capacity to detect and respond to new zoonotic threats before they become more widespread.

## An Historical View of Flu

The H1N1 virus first emerged around the time of the pandemic of “Spanish flu,” which infected one-third of the world’s population and killed up to 100 million people between 1918 and 1920. During the later stages of that pandemic, farmers noticed that pigs also were getting sick with the malady scientists at the time called “hog flu.” Hog flu was reported intermittently in the Midwestern United States long after the human pandemic ended. In 1930, Richard E. Shope, while working at The Rockefeller Institute for Medical Research, identified the cause of the animal illness: the influenza virus now known as H1N1, named in reference to its hemagglutinin (H) and neuraminidase (N) surface proteins.

Jürgen A. Richt, a distinguished professor at Kansas State University College of Veterinary Medicine, and other experts believe that after 1918, H1N1 established itself in pigs, which unlike monkeys, mice, or ferrets, can survive the infection. Scientists can’t conclusively say if humans first infected pigs with the H1N1 virus or vice versa, Richt says. But what is clear, he adds, is that pigs have been a reservoir for the virus ever since.

As a group, H1N1 viruses are common in pigs, which typically experience minor flu symptoms when infected. Studies published by Canadian experts, such as a report by Zvonimir Poljak et al. in the January 2008 issue of the *Canadian Journal of Veterinary Research*, indicate that swine flu is more likely to be present and to persist in larger farms with higher pig densities. This suggests the

CAFO environment may be more likely than smaller farms to facilitate the evolution of novel strains. CAFO workers can also pick up H1N1 infections and experience a range of symptoms depending on their own immunity, says Gray. In humans, what makes novel H1N1 unique is its remarkable and still mysterious capacity for person-to-person transmission. “The strain has a unique presentation of antigens [surface proteins that evoke immune response],” Gray says. “Most people have never been exposed to anything like it before. We’re still not sure why it transmits so readily from one person to another; this is the subject of a lot of research.”

Novel H1N1 contains genes from North American and Eurasian swine influenza viruses, explains Carolyn Bridges, associate director for epidemiologic science in the Influenza Division of the Centers for Disease Control and Prevention (CDC). These 2 viral strains could have mixed in either pigs or humans, Bridges suggests, or less likely in other animals such as wild birds. Gray says the strain could even have been created inadvertently by scientists engaged in recombinant viral research with H1N1. But it’s also biologically plausible, he adds, that the virus could have evolved in a CAFO somewhere in the world.

One thing that’s certain is that no one can say exactly where the pandemic strain evolved, says Silbergeld. Moreover, its origins can’t be determined retrospectively, given how fast influenzas mutate as they pass from host to host.

In the absence of publicly available sampling data, speculation about the origins of the current pandemic have run rampant. In April 2009, bloggers including Tom Philpott, writing for the environmental website Grist, and David Kirby, for the Huffington Post, created a stir when they pointed a finger at Mexican CAFOs run by Smithfield Foods subsidiary Granjas Carroll de México. Each year these CAFOs raise some 950,000 hogs on 16 farms along the border of the Mexican states of Veracruz and Puebla. One such CAFO lies about 5 miles outside the town of Perote, home to the pandemic’s first reported case. Philpott cited as evidence newspaper interviews with Perote residents, who claimed infectious pollution from the CAFO had sickened the 5-year-old victim, a boy who later recovered. Those claims were never scientifically confirmed, however, and Mexican officials later identified another case from a different part of the country who could have been infected as early as February.

## Novel H1N1 Raises the Stakes

The novel H1N1 episode comes on the heels of a protracted backlash against the CAFO industry, which has been blamed by critics for a host of environmental ills. Swine CAFOs generate vast amounts of fecal waste, stored in onsite lagoons that can breach and pollute local watersheds during heavy rains. The facilities emit a piercing odor that can be detected up to 6 miles from its source if not managed properly.

Steven Wing, an associate professor of epidemiology at the University of North Carolina at Chapel Hill, reported in the March 2000 issue of *EHP* that CAFO odors can evoke emotional distress and headaches among local residents. “The biggest differences between CAFO-exposed communities and matched controls are found in quality-of-life measures,” he says of his findings. “These are indicated by how often local residents report that they can’t open windows or go outside even in nice weather.”

According to Wing, the ammonia-laden airborne emissions released by CAFOs are also linked to asthma, mucous membrane irritation, and other respiratory sysmptoms. The facilities have further been implicated in the emergence of antibiotic-resistant bacteria.

In the face of harsh criticism, the industry claims its practices are unfairly maligned. “The story our critics seldom tell is that all our farms are permitted by states or the federal government and subject to regular inspections,” says Dennis Treacy, Smithfield Foods’ vice president of environmental and corporate affairs. “We also require that all our farms go through stringent environmental management programs on top of permit requirements.”

But a closer look reveals that CAFOs fall through regulatory cracks when it comes to sampling for novel viruses that could make people sick. Bridges explains that producers have little incentive to test for swine influenzas, in part because they aren’t included on a list of 150 “reportable illnesses” that, when detected, must be documented with the World Organisation for Animal Health (OIE). Based in Paris, the OIE is the veterinary disease counterpart to the World Health Organization.

Kay Johnson Smith, executive vice president with the Animal Agriculture Alliance, an industry-funded educational group, points out the OIE doesn’t consider swine influenzas reportable because it views them as routine infections in animal agriculture. “H1N1 is a standard swine flu,” she says, “and therefore, like other flus such as standard avian or equine flu, they aren’t reportable as emerging diseases.”

Novel H1N1 also is not required to be reported to OIE, although in a 21 July 2009 editorial posted on the organization’s website, director general Bernard Vallat “strongly advises” members to report animal cases of the disease. As of 29 July 2009, only 4 swine herds had been shown to be infected with novel H1N1: 1 in Alberta, Canada; 1 in Québec, Canada; and 2 in Argentina’s Buenos Aires Province. But public health experts have been unable to look for the new strain in large-scale industrial farms in part because the pork industry won’t allow them to, according to Gray. He says pork producers could face global trade sanctions and economic losses should the strain be revealed in its animals. The Alberta herd was in fact destroyed without compensation to its owner when the infection was discovered.

“There’s considerable aversion among swine producers to even test for the pandemic strain,” Gray claims. “They worry they’d have to destroy their animals and lose that income.” Moreover, countries that report food animal diseases to the OIE can suffer trade sanctions to protect importers from foreign infections.

As far as international surveillance goes, Vallat wrote in his 21 July 2009 editorial that “OIE has called upon the expertise from [collaborating laboratories and research centers] to publicly share genetic sequences of influenza identified in swine in full transparency, to facilitate the early preparation of human and animal vaccines if needed. The OIE will continue to draw the attention of its Members and of the public at large that all potential zoonotic diseases must be controlled by strengthening the Veterinary Services in order to improve the early detection, rapid response, surveillance and reporting capabilities of animal diseases including zoonoses in all countries, regardless of their level of development and trade potential.”

In May 2009, the U.S. Department of Agriculture (USDA) announced a $1.5 million surveillance program to look for novel flu strains in pigs. But according to USDA spokeswoman Angela Harless, this program, which was funded by the CDC in September of 2008, will sample only sick pigs submitted voluntarily by swine growers to government and private labs, in addition to herds exposed to the novel H1N1 strain by sick CAFO workers.

“USDA continues to encourage swine growers to report sick pigs to their herd veterinarian, state animal health official, or area vet in charge,” she says. “If swine herds test positive for novel H1N1, they will be closely monitored and allowed to move in commerce once they have recovered.”

But Richt asserts that without more industry cooperation, the USDA’s surveillance program is “dead in the water.” In other words, he explains, producers won’t submit their animals for analysis without a guarantee of indemnification, meaning economic protection to recover losses should the virus be discovered.

## CAFO Employees: A Crucial Link

Gray says he welcomes the new USDA surveillance program, but he worries that it lacks a crucial element: The protocol doesn’t include CAFO workers, who can infect pigs with influenza and also be infected by them. When zoonotic flu viruses jump from species to species, they can pick up new mutations and reassort into novel strains that might be unrecognizable to both animal and human immune systems, Gray explains. “If we want to detect novel viruses from the human–animal interface, then we also need to study the workers,” he emphasizes.

The agency charged with ensuring worker safety is the Occupational Safety and Health Administration (OSHA). However, OSHA typically exempts facilities with fewer than 11 employees from routine inspection unless otherwise requested by employees or other agencies. Yet, like many other modern production facilities, CAFOs are largely automated, so a typical factory farm housing 2,000 sows requires a crew of just 7 people, according to Don Butler, director of government relations and public affairs for Murphy-Brown, the livestock production subsidiary of Smithfield Foods. And Wing adds that CAFOs in some regions are often staffed by black and Hispanic workers who might fear racial harassment for reporting safety infractions to OSHA, as well as low-income workers of all races who worry about keeping their jobs in the industry and access to health care, housing, and other services provided by their employers.

When asked how OSHA regulates zoonotic disease risk at CAFOs, a spokesman at the agency said its purview applies exclusively to bloodborne pathogens via the Bloodborne Pathogens Standard (29 CFR 1910.1030), which excludes respiratory infections such as swine flu. OSHA considers the risk of zoonotic disease only in terms of health care workers involved in responding to a pandemic. But the spokesman could not confirm whether a CAFO had ever been cited for violation of the Bloodborne Pathogens Standard. “The information on inspections in our database is not detailed enough to make this determination,” he says.

To develop its CAFO inspection strategies, OSHA draws on research generated by the National Institute for Occupational Safety and Health (NIOSH). According to public health officer Frank Hearl, NIOSH’s CAFO research activities are proceeding on 2 fronts, one addressing noise exposure (a common occupational hazard for swine farmers) and another quantifying airborne microbial levels at CAFOs. This latter activity, Hearl says, is managed by an extramural grant awarded to Norman Pace, a distinguished professor in the Department of Molecular, Cellular, and Developmental Biology at the University of Colorado, Boulder. When asked about his research, Pace said it doesn’t deal with zoonotic illnesses. A possible exception, he says, is Johne disease, a wasting affliction in cattle that is suspected by some to be a cause of human Crohn disease. That proposed link has never been scientifically proven.

Speaking for the private sector, Mary Battrell, a veterinarian with Murphy-Brown, says the company has no formal flu surveillance plan for its workers, although it does require that they get a flu shot every year.

Gray and Silbergeld believe CAFO workers and veterinarians should be seen as high-priority groups for influenza surveillance and vaccination. These groups, Gray says, experience the biggest risk factor for zoonotic infection through their routine and intensive exposure to food animals. They also serve as “bridge populations” that can transfer these infections from animals to the wider public, he says.

In the 30 May 2007 issue of *Vaccine*, Gray and colleagues estimated the current U.S. swine and poultry CAFO workforce at about 54,000 workers—the number is difficult to gauge, they explained, because these workers have no unifying membership organization. “Considering other high-risk groups in the U.S. national plans targeted for special access to pandemic vaccines and antivirals (e.g., 8–9 million U.S. medical and public health workers), the number of swine and poultry workers is relatively few,” the authors wrote. “Hence, the investment in protecting them is relatively small and very likely cost-effective.”

Building on a growing body of evidence, Gray’s research strongly suggests that CAFO workers and veterinarians can infect other people with H1N1 viruses. In a 2-year prospective study of 803 rural Iowans, published in *Emerging Infectious Diseases* in December 2007, he found that CAFO workers were 50 times more likely to have elevated H1N1 antibodies than nonexposed controls. Equally important, their spouses were 25 times more likely to harbor these antibodies, reflecting how the viruses can jump from farm workers to their intimate contacts. Similarly, in work published 15 May 2009 in the *Journal of the American Veterinary Medical Association*, Gray and coauthor Whitney S. Baker reported that 84% of 44 seroepidemiologic studies reviewed identified an increased risk of zoonotic pathogen infection among veterinarians.

Beyond surveying workers directly, Gray adds, academic researchers should also sample CAFO environments. “We need to look for viruses in the air, swab surfaces, and follow the pigs and workers and figure out how the viruses jump species,” he says. “It’s important to find out how these viruses move and persist in CAFO environments from one pig cohort to the next. If we find a high prevalence of viral infection with a given strain in January, why do we see it again the next January if the pigs live only six months before slaughter? We need to study the pigs, the workers, and the environment to understand how the viruses get around, and what sort of interventions we can take to limit transmission.” Silbergeld recently submitted a grant proposal to the NIEHS to further study zoonotic pathogens including their persistence in CAFO environments and nearby communities.

## Relying on Biosecurity

A crucial question is whether other, potentially more lethal influenzas could emerge from CAFOs, assuming these facilities allow new strains to evolve with unprecedented speed and efficiency. According to Andrew Pekosz, an associate professor of microbiology and immunology at the Johns Hopkins Bloomberg School of Public Health, newly virulent strains emerge randomly, by chance. By concentrating so many viruses in one place, he explains, CAFOs increase the frequency at which more dangerous strains might appear. “This is all a numbers game,” he says. “The more variants you’re exposed to, the more likely it is that you’ll be exposed to one with altered properties that allows for infection of a new host.”

Given that threat, the industry relies on biosecurity measures to prevent pathogens from getting into or out of CAFOs. Johnson Smith says biosecurity protocols are company-specific and can vary by facility. In general, she says, CAFOs require employees to shower before they enter and leave the workplace, and to wear protective clothing that covers their hair, shoes, and clothes. “Only authorized personnel are allowed to enter the animal facilities, and authorization is required for anyone else,” she says. “Some people say we have something to hide, but these security measures protect animal health and safety, as well as food safety. They are in place to help prevent the spread of disease from humans to animals.”

Butler says that in addition to these measures, the company requires employees to avoid a CAFO for at least 4 days if they’ve been out of the country to avoid introducing exotic diseases. “And our farms are located in rural areas, which tend to be sparsely populated with wide-open spaces,” he adds. “They’re not immediately adjacent to residential areas.”

But in practice, CAFO workers don’t always wear the recommended protective gear, according to multiple scientists who have studied such facilities. In addition, Wing counters there are other routes by which pathogens could get into or out of CAFOs regardless of these biosecurity protocols. For instance, hog waste stored in onsite lagoons can spill into streams or penetrate into groundwater, he says. Untreated hog wastes are also routinely sprayed on crop fields as fertilizer. And, although not a standard industry practice, anecdotal evidence suggests hog carcasses are sometimes loaded into dumpsters, raising the possibility that vectors could carry hog pathogens to surrounding environs.

The degree to which hog waste might be pathogenic is unknown. Echoing conclusions reached by others, Wing says there’s no evidence to suggest that communities living near CAFOs have elevated rates of infectious illness. Kelley Donham, a professor of occupational and environmental health at the University of Iowa College of Public Health, adds that influenza in particular doesn’t survive well in the environment. “It’s a respiratory virus,” he says. “It’s always looking for respiratory epithelial cells, so it doesn’t seem plausible to suggest that it could be transmitted through waste.”

Still, Donham says other zoonotic pathogens in hog waste—particularly bacterial agents including *Salmonella*, *Leptospira*, and some infectious strains of *Escherichia coli*—could travel downwind as spray aerosols and theoretically infect local populations.

Moreover, he says, air- and waterborne CAFO emissions are often contaminated with antibiotic-resistant microbes such as methicillin-resistant *Staphylococcus aureus* (MRSA). In an article published 23 January 2009 in *PLoS ONE*, Tara C. Smith of the University of Iowa College of Public Health and colleagues reported finding pig-specific MRSA strains among farmworkers and swine in Iowa and Illinois. Antibiotic uses in agriculture exceed human clinical use by roughly eightfold, Silbergeld says, such that industrial-scale farms compete with hospitals as a major source of the antibiotic resistance that plagues medical care today [for more information, see “The Landscape of Antibiotic Resistance,” *EHP* 117:A244–A250 (2009)].

## Conflict of Interest?

Meanwhile, Robert Martin, senior officer with the Pew Environment Group in Washington, DC, is concerned that competing financial interests may be partly to blame for the current lack of data and regulation. “Even the best scientists seem loathe to say anything against the industry,” he says. “With the decline in public research funding, it’s industrial animal agriculture that pays for virtually all the animal sciences research going on at land-grant universities today.”

Martin directed the Pew Commission on Industrial Farm Animal Production, which released its final report, *Putting Meat on the Table: Industrial Farm Animal Production in America*, in April 2008. In preparing that report, he says, “We looked at that close relationship, and it seemed to us that the research is biased to generate more industry profit. Many academic researchers are concerned about alienating their primary source of research dollars—i.e., the industrial animal sector—and that makes them leery of looking at industry problems with an open mind.”

“We agree there should be more public funding, but since our government and Congress are not allocating enough money for this purpose, someone has to pay to have research done,” retorts Johnson Smith. “Can we truly afford as a nation to not do research? Who else will pay for agricultural research besides those with a vested interest in advancing and improving current practices?”

These points lead to new questions: Who should decide how current practices are advanced and improved? And what constitutes “advancement” and “improvement”? Answers to these questions devolve quickly into subjective and wildly divergent views about how food production should be managed, yet this vastly complex topic defies simple solutions.

Industry argues the trend toward CAFO-dominated animal agriculture is safer than more traditional farming, wherein livestock spend much of their time outside, exposed to inclement weather and viruses from other animals, including wild birds. Among other preventive measures, says Johnson Smith, pork producers raise their stock in enclosed buildings with bird wire to prevent birds from entering the facility, and pig feed is stored in enclosed bins that prevent contamination with bird droppings.

In another example of how traditional farming comes with its own infectious disease risks, Smith says infection with *Trichinella spiralis* (the source of the dangerous foodborne human disease trichinosis) is much rarer in CAFO-raised swine than in pigs raised outdoors, which are more likely to eat a diet of swill and the occasional rat or wild animal carcass. “The real question,” Smith says, “is how much of a threat do these CAFO facilities really pose, and what are the tradeoffs we have to accept for having affordable protein sources?”

Yet CAFOs can be shocking to the senses and capable of evoking highly charged public reactions. These reactions routinely put the industry in a defensive posture as it attempts to sway opinion in its favor with communication strategies routinely dismissed by critics as spin. Extracting accurate information in this embattled context is challenging at best—or, some say, impossible.

As with other complex topics, nearly every significant aspect of CAFO production can be viewed from multiple perspectives. But perhaps this much is clear: the current pandemic shows that viruses of animal origin can pose a substantial human health threat. And if CAFOs were to accelerate the evolution of these viruses, Gray says, then the public has a right to know how those viruses evolve and what steps can be taken to limit their spread. “If we find something new,” he says, “we need to heighten surveillance to track it—not sit on it and pretend nothing’s happening until the problem explodes.”

## Figures and Tables

**Figure:**
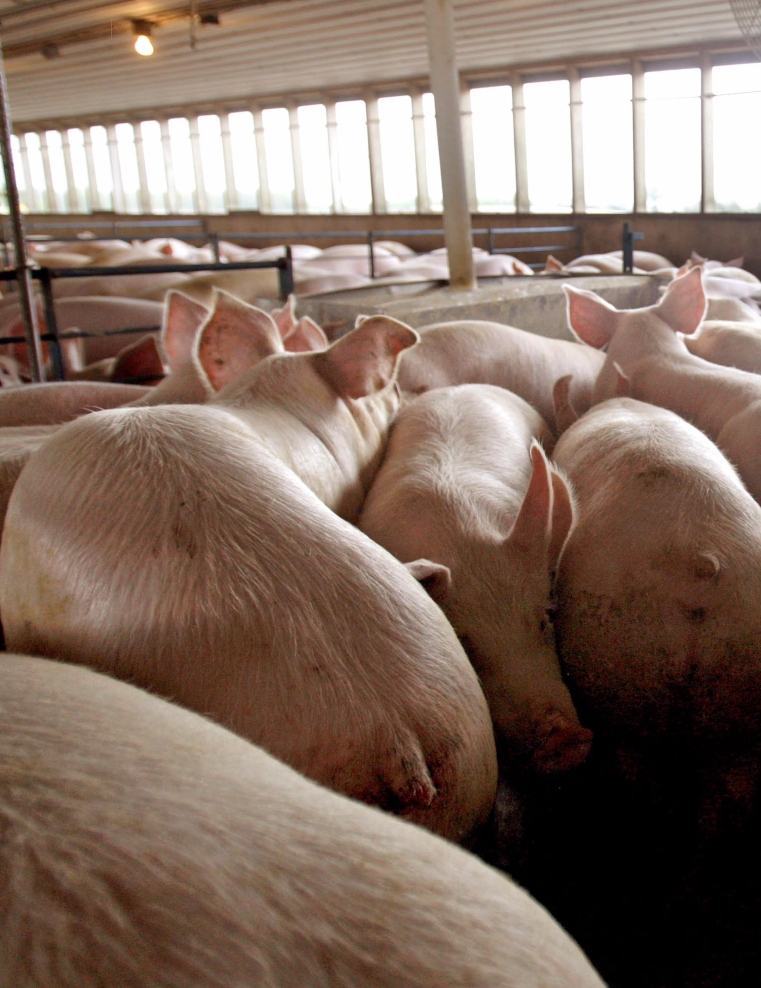
A hog farm in Auxvasse, Missouri, 30 April 2009

**Figure:**
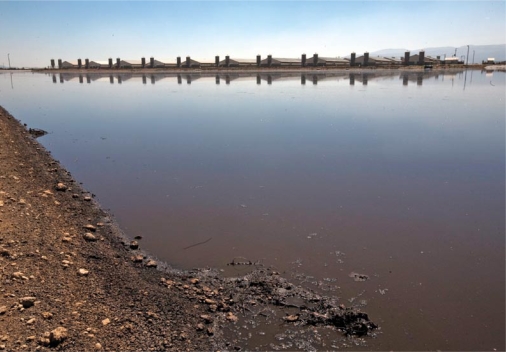
**The waste lagoon for a CAFO near Perote, Mexico, 1 May 2009** According to early media reports, residents of Perote believe waste from nearby swine CAFOs run by Granjas Carroll de México was the source of the novel H1N1 that infected a local child, the first reported case in the pandemic. Earlier cases have since been identified elsewhere, and it is impossible to say now where or how the pandemic began.

**Figure:**
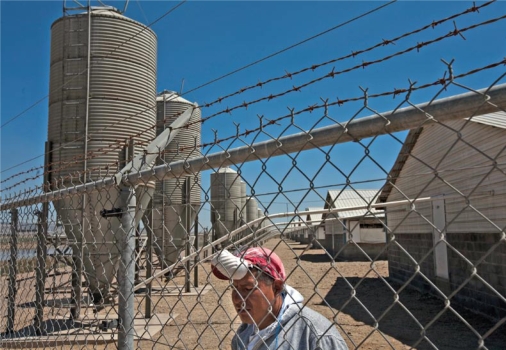
**An employee at a CAFO run by Granjas Carroll de México, 1 May 2009** It takes only a few workers to run a facility housing thousands of animals. Automated systems deliver food and water at regular intervals, and waste is flushed periodically. Workers monitor the automated equipment and check the animals for any health or behavior problems.

**Figure:**
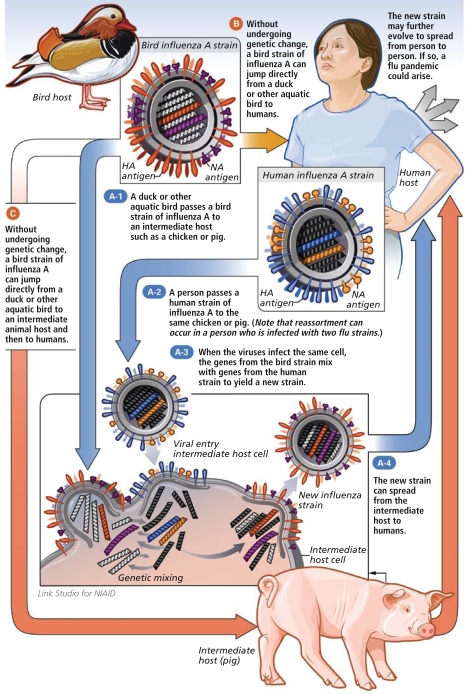
How Novel Influenza Strains Evolve

**Figure:**
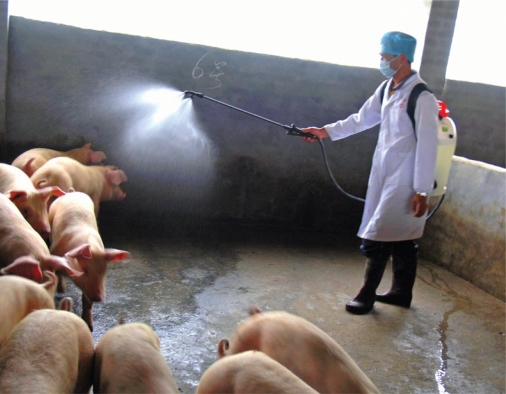
**A Chinese farmer sprays disinfectant on pigs, 29 April 2009** The World Organisation for Animal Health (OIE)—the veterinary medicine counterpart to the World Health Organization—does not require but “strongly advises” farmers to report cases of novel H1N1 detected in livestock and to intensify their surveillance for potential infections. In a 21 July 2009 editorial, OIE director general Bernard Vallat wrote, “The creation of a vaccine directed against this virus in pigs could be one of the solutions to control the disease in animals if the number of infected herds becomes excessive, because of the potentially growing number of animals infected by humans in the context of the human pandemic.”

**Figure:**
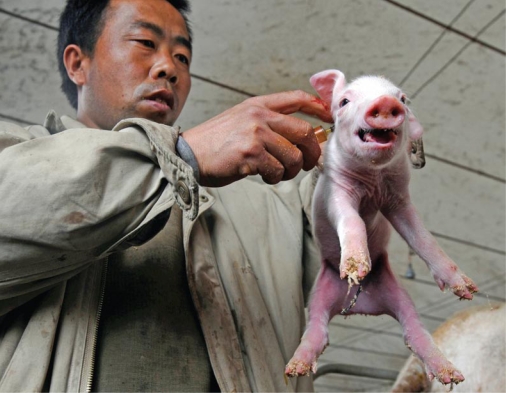
A Chinese piglet is vaccinated soon after birth, 30 April 2009

